# Supplementary material for: Comparative Transcriptomics Reveals Novel and Differential Circular RNA Responses Underlying Interferon-Mediated Antiviral Regulation in Porcine Alveolar Macrophages
Source: Viruses. 2025 Sep 27;17(10):1307. doi: 10.3390/v17101307 (PMC12567601; doi:10.3390/v17101307)
Supplement: Supplementary file 1 [file viruses-17-01307-s001.zip › Supplementary Material.pdf]

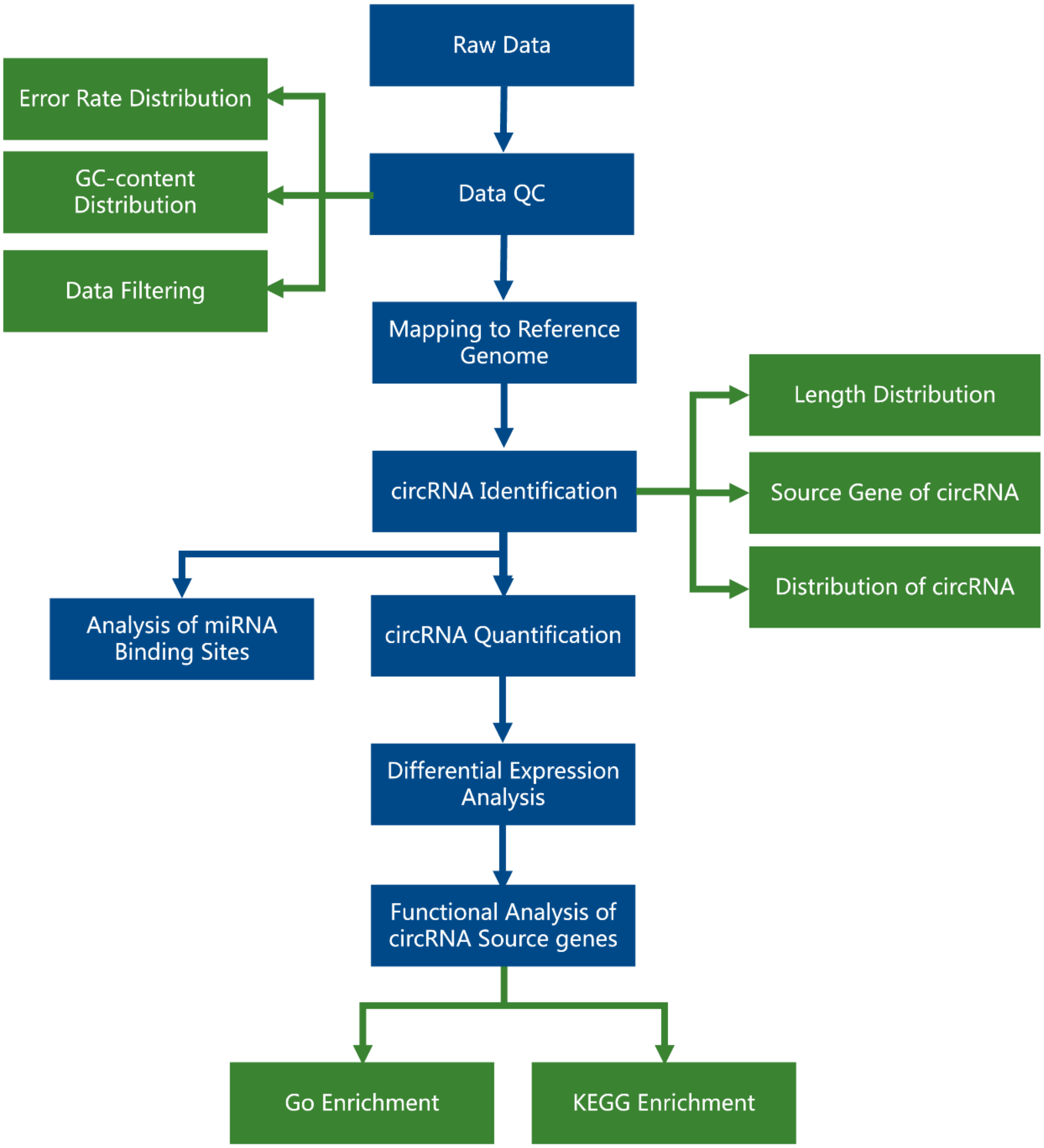

Figure S1. The workflow of whole transcriptomic analysis (WTS) for circRNA profiling in porcine alveolar macrophages. Primary alveolar macrophages were cultured/treated, and high-quality RNA samples were obtained for sequencing library preparation and sequencing using an Illumina platform. Major steps in the sequential data analysis are shown in blue, while itemized sub-steps are shown in green. The workflow includes raw data quality control, mapping to the reference genome, circRNA identification and quantification, differential expression analysis, functional enrichment analysis of circRNA source genes, and prediction of miRNA binding sites.

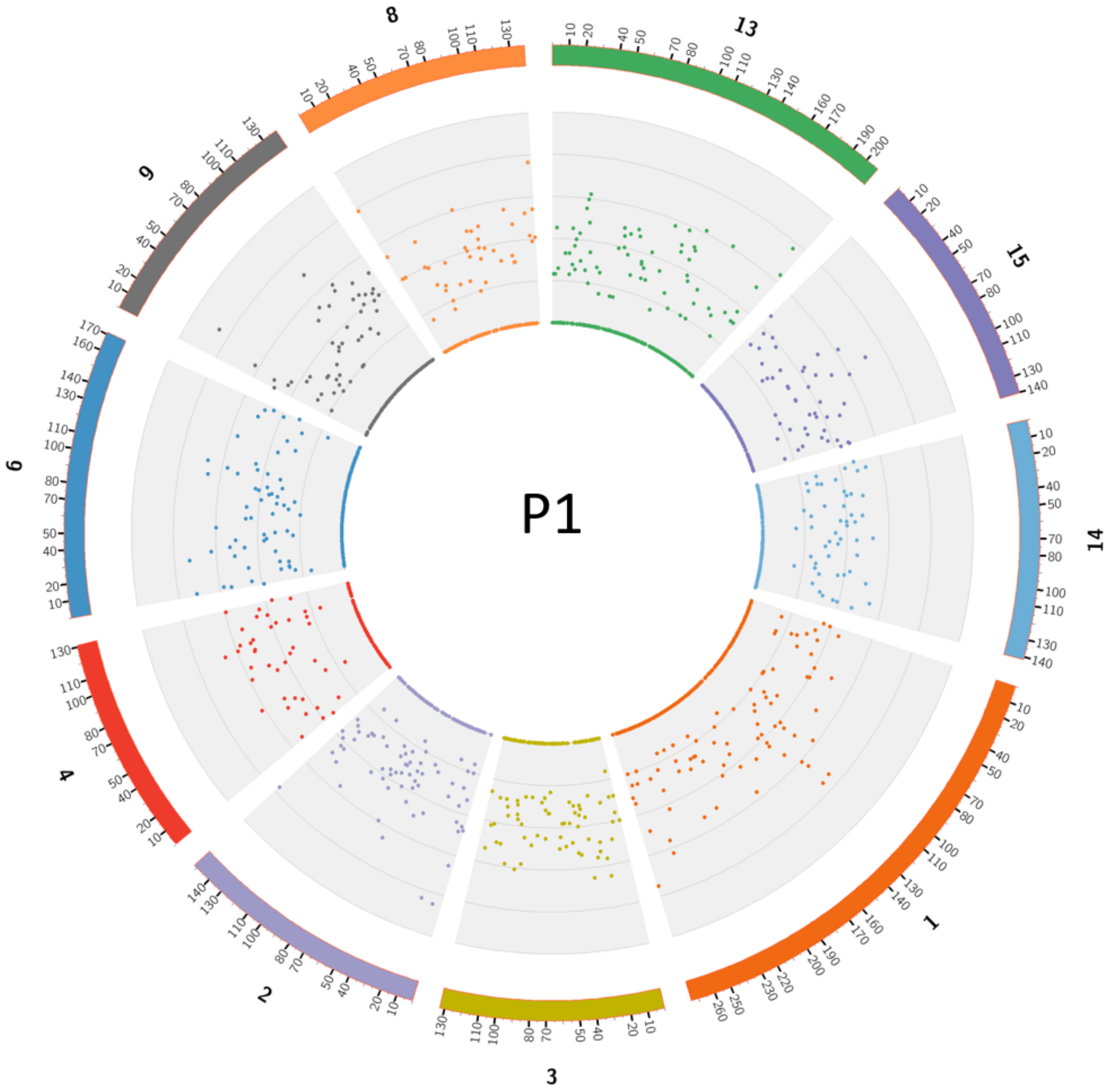
P1
8
13
15
14
1
3
2
4
6
9

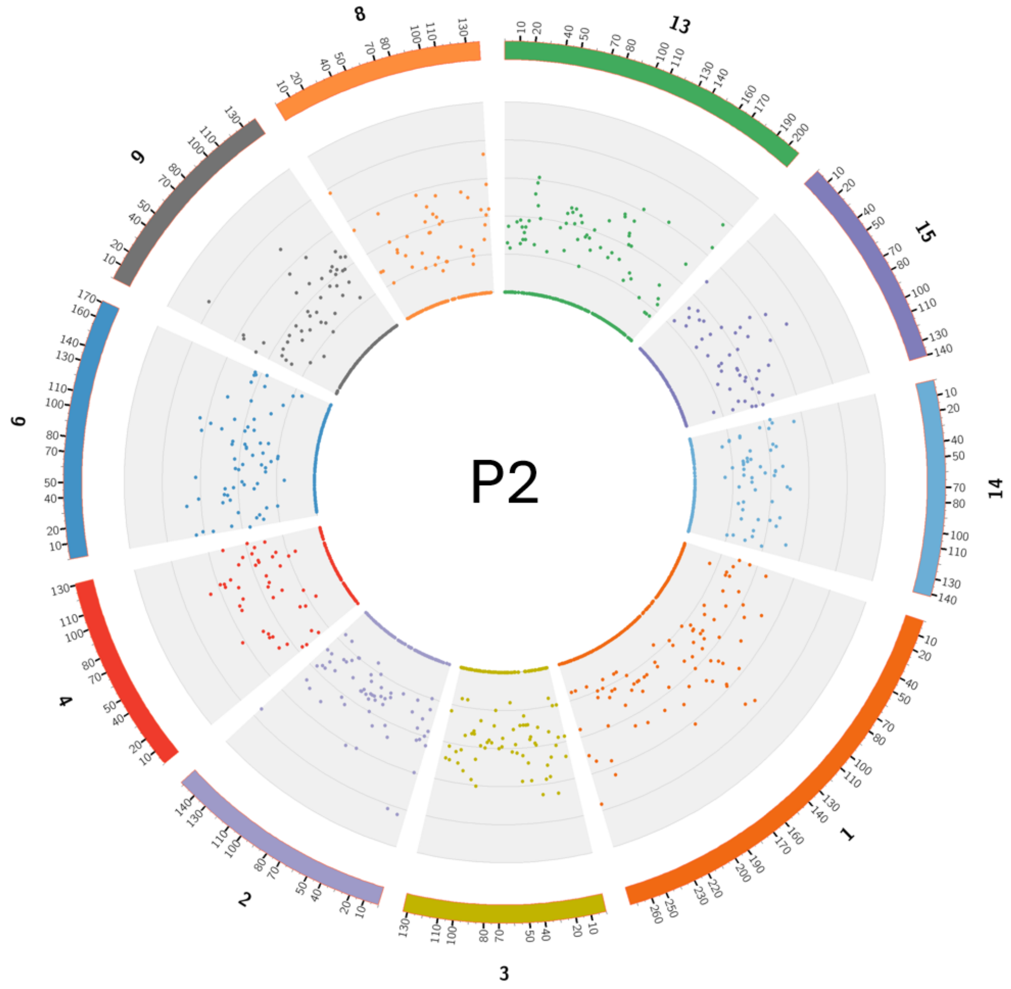
P2
8
13
15
14
1
3
2
4
6
9

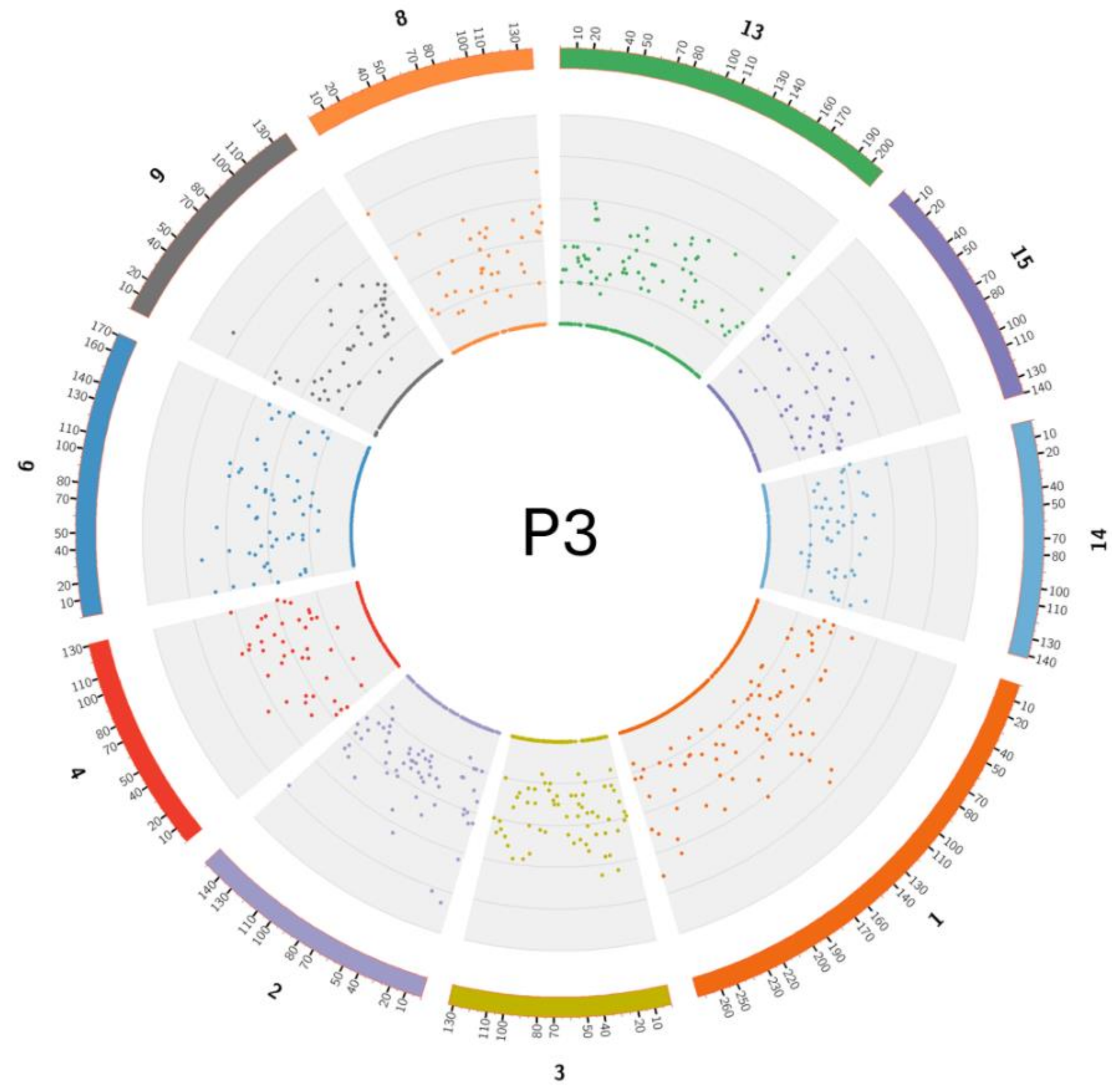

Figure S2. The density of total mapped reads in each chromosome was calculated, and 10 chromosomes or scaffolds were selected for cross-sample comparison (P1, P2, P3)

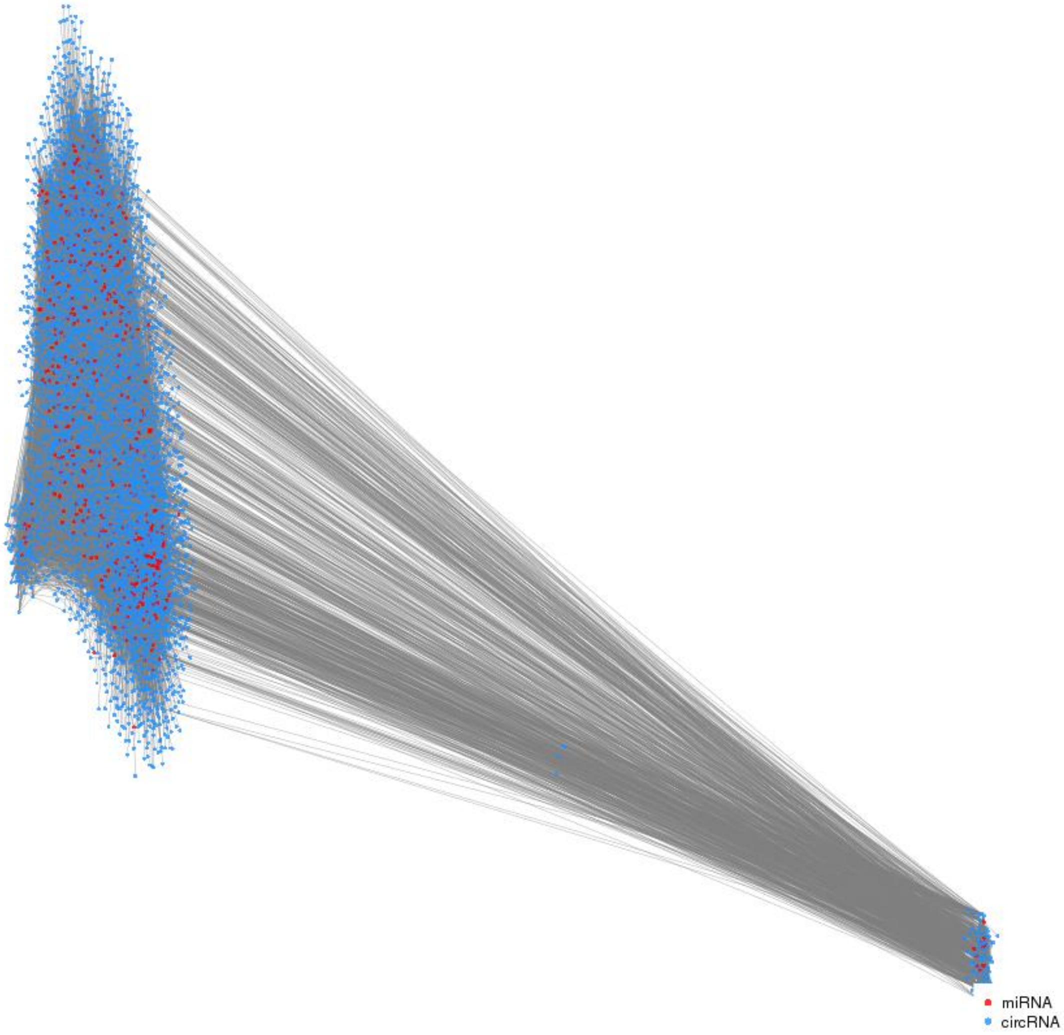

Figure S3. Comprehensive circRNA–miRNA interaction network generated from miRanda predictions. Each blue node represents a circRNA, and each red node represents a miRNA. Gray edges indicate predicted binding interactions between circRNAs and miRNAs.

Table S1. Software used in the analysis.

Software name	Details	Version	Parameter
bowtie2	Mapping	2.2.3	-very-sensitive
HTseq-count	Distribution	0.6.0.	-m union -s reverse
circos	Distribution	0.62-1	default
blastx	NR database mapping	2.2.28	default
ng_qc	Quality control	1.0	default
hisat2	Mapping	2.0.4	--dta --rna-strandness RF
RSeQC	Strand analysis	2.3.9	default
samtools	BAM, SAM processing	0.1.18	default
bedtools	Reference processing	2.19.1	default
BWA-mem	mapping	0.7.8-r455	mem -t 4 -T 19
CIRI2	Identification of circRNAs	2.0.5	default
CIRI AS	Identification of circRNAs	1.2	default
find_circ	Identification of circRNAs	1.2	default
kobas	KEGG enrichment	3.0	Corrected P-Value<0.05
GOSeq,topGO	GO enrichment	2.12	EnrichmentMethod: Wallenius hmmscan Padjust: BH Corrected P-Value<0.05
psRobot	miRNA targets(for plants)	1.2	-p 1
miRanda	miRNA targets(for animals)	3.3a	-sc 140 -en -10 -scale 4 –strict
DEGSeq	Differential expression analysis(samples without biological replicates)	1.12.0	log2foldchang>1 or log2foldchang< -1 && qvalue<0.05
DESeq2	Differential expression analysis(samples with biological replicates)	1.6.3	padj<0.05
DeepIRES	IRES prediction	1.0	default
DeepCIP	IRES prediction	2.22	-b 16, -c 0.5, -m 0
deepSRAMP	m6A prediction	1.0	3, 2
